# 10-(4-Chloro­phen­yl)-14a-hy­droxy-12-methyl-8,9,9a,10,12,13,14,14a-octa­hydro-5*H*-10a,14-methano­indeno­[2′,1′:4,5]azepino[3,4-*b*]pyrrolizine-5,15(7*H*,11*H*)-dione

**DOI:** 10.1107/S1600536813034107

**Published:** 2013-12-24

**Authors:** R.A. Nagalakshmi, J. Suresh, K. Malathi, R. Ranjith Kumar, P. L. Nilantha Lakshman

**Affiliations:** a Department of Physics, The Madura College, Madurai 625 011, India; b Department of Organic Chemistry, School of Chemistry, Madurai Kamaraj University, Madurai 625 021, India; c Department of Food Science and Technology, University of Ruhuna, Mapalana, Kamburupitiya 81100, Sri Lanka

## Abstract

The asymmetric unit of the title compound, C_26_H_25_ClN_2_O_3_, contains two independent mol­ecules (*A* and *B*). The conformation of the two mol­ecules differs essentially in the dihedral angle involving the two benzene rings. They are inclined to one another by 52.47 (10) in *A* and by 31.75 (11)° in *B*. In both mol­ecules, the six-membered piperidin-3-one rings have chair conformations. In mol­ecule *A*, all four five-membered rings have twist conformations. In mol­ecule *B*, only three of the four five-membered rings have twist conformations. The fourth, of the inden-1-one moiety, has an envelope conformation with the spiro C atom, bonded to the N atom of the pyrrolidine ring, as the flap. A weak intra­molecular O—H⋯N hydrogen bond occurs in each independent mol­ecule while a C—H⋯O inter­action is also observed in mol­ecule *A*. In the crystal, pairs of O—H⋯O hydrogen bonds link the mol­ecules, forming inversion dimers with graph-set motif *R*_2_^2^(12). These dimers are further inter­connected by C—H⋯O and C—H⋯π inter­actions, forming a three-dimensional network.

## Related literature   

For the importance of pyrrolizine derivatives, see: Anderson & Corey (1977 ▶); Makoni & Sugden (1980 ▶); Barsoum & Nawar (2003 ▶); Abbas *et al.* (2010 ▶). For the importance of piperidines, see: Rubiralta *et al.* (1991 ▶); Pinder (1992 ▶); Michael (2001 ▶). For puckering parameters, see: Cremer & Pople (1975 ▶).
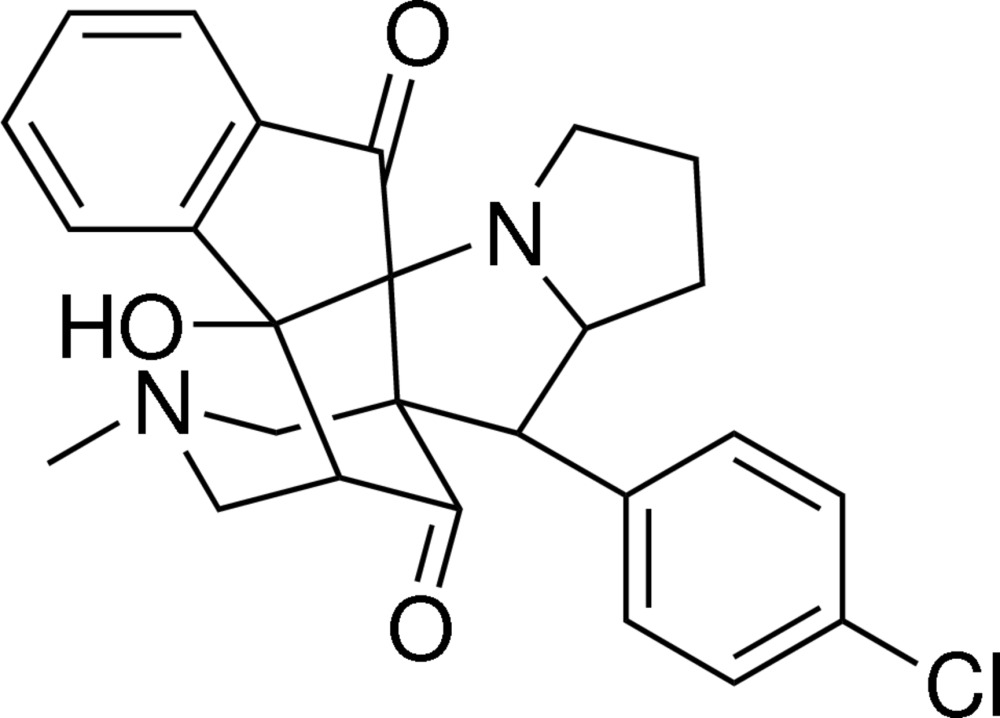


## Experimental   

### 

#### Crystal data  

C_26_H_25_ClN_2_O_3_
*M* *_r_* = 448.93
Monoclinic, 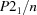

*a* = 13.5839 (4) Å
*b* = 11.3764 (4) Å
*c* = 28.5382 (9) Å
β = 95.120 (2)°
*V* = 4392.6 (2) Å^3^
*Z* = 8
Mo *K*α radiation
μ = 0.21 mm^−1^
*T* = 293 K
0.21 × 0.19 × 0.18 mm

#### Data collection  

Bruker Kappa APEXII diffractometer
Absorption correction: multi-scan (*SADABS*; Sheldrick, 1996 ▶) *T*_min_ = 0.967, *T*_max_ = 0.974
85408 measured reflections
10365 independent reflections
7088 reflections with *I* > 2σ(*I*)
*R*_int_ = 0.041

#### Refinement  

*R*[*F* ^2^ > 2σ(*F* ^2^)] = 0.046
*wR*(*F* ^2^) = 0.123
*S* = 1.01
10365 reflections
591 parameters
H atoms treated by a mixture of independent and constrained refinement
Δρ_max_ = 0.33 e Å^−3^
Δρ_min_ = −0.38 e Å^−3^

### 

Data collection: *APEX2* (Bruker, 2004 ▶); cell refinement: *SAINT* (Bruker, 2004 ▶); data reduction: *SAINT*; program(s) used to solve structure: *SHELXS97* (Sheldrick, 2008 ▶); program(s) used to refine structure: *SHELXL97* (Sheldrick, 2008 ▶); molecular graphics: *PLATON* (Spek, 2009 ▶); software used to prepare material for publication: *SHELXL97*.

## Supplementary Material

Crystal structure: contains datablock(s) global, I. DOI: 10.1107/S1600536813034107/ds2236sup1.cif

Structure factors: contains datablock(s) I. DOI: 10.1107/S1600536813034107/ds2236Isup2.hkl

Additional supporting information:  crystallographic information; 3D view; checkCIF report

## Figures and Tables

**Table 1:** Hydrogen-bond geometry (Å, °) *Cg*1 is the centroid of the C21*A*–C26*A* ring.

*D*—H⋯*A*	*D*—H	H⋯*A*	*D*⋯*A*	*D*—H⋯*A*
O1*A*—H1*A*⋯N2*A*	0.82	2.20	2.705 (2)	120
O1*B*—H1*B*⋯N2*B*	0.82	2.16	2.689 (2)	123
C8*A*—H8*A*⋯O2*A*	0.98	2.57	3.190 (2)	122
O1*B*—H1*B*⋯O3*B* ^i^	0.82	2.51	3.147 (2)	135
C1*A*—H3⋯O3*B* ^ii^	0.96 (3)	2.59 (3)	3.164 (3)	118 (2)
C1*A*—H2⋯*Cg*1^iii^	1.02 (3)	2.70 (3)	3.648 (3)	155 (2)

Symmetry codes: (i) 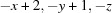; (ii) 
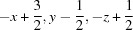
; (iii) 
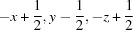
.

**Fig. 1.**
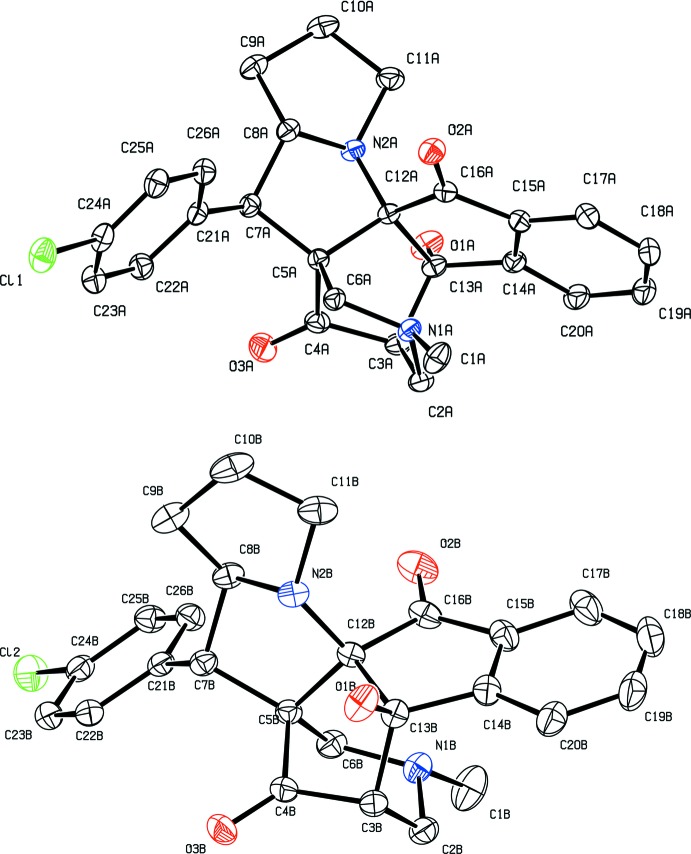
The molecular structure of (I), showing 20% probability displacement ellipsoids and the atom-numbering scheme. H-atoms are omitted for clarity.

**Fig. 2.**
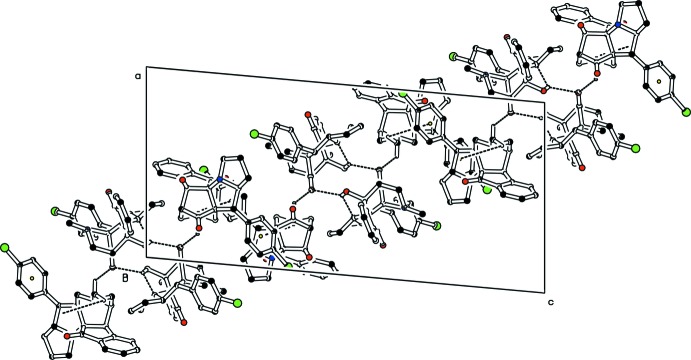
Partial packing diagram.

## supplementary crystallographic information

### 1. Comment

Pyrrolizine derivatives have antimicrobial (Barsoum & Nawar, 2003), anti- inflammatory (Abbas *et al.*, 2010) and antileukemic (Anderson & Corey, 1977) activities. These derivatives are used as inhibitors of blood platelet aggregation (Makoni & Sugden, 1980), Functionalized piperidines are familiar substructures found in biologically active natural products and synthetic pharmaceuticals (Michael, 2001; Pinder, 1992; Rubiralta *et al.*, 1991). Against this background and to ascertain the molecular structure and conformation, the X-ray crystal structure determination of the title compound has been carried out.

The asymmetric unit of the title compound (Fig 1) C_37_H_28_N_2_O_3_, comprises two independent molecules. In the pyrrazoline ring system, the pyrrolidine rings (N2/C8/C7/C5/C12) adopt twisted conformations with the puckering parameters q_2_ = 0.4373 (18) Å and Φ_2_ = 13.0 (2) ° and q_2_ = 0.4096 (19) Å and Φ_2_ = 193.9 (3)° (Cremer & Pople, 1975), in molecule A and in molecule B respectively. The pyrrolidine rings (N2/C8—C11) adopt twisted conformation with the puckering parameters q_2_ = 0.453 (2) Å, Φ_2_ = 194.8 (3) ° and q_2_ = 0.461 (2) Å, Φ_2_ = 10.9 (3) ° (Cremer & Pople, 1975) respectively for molecule A and molecule B. The cyclopentane ring (C12A—C16A) in molecule A adopts a twisted conformation with the puckering parameters q_2_ = 0.0775 (19) Å and Φ_2_ = 18.0 (1) ° (Cremer & Pople, 1975). The cyclopentane ring (C12B—C16B) in molecule B adopts an envelop conformation with the puckering parameters q_2_ = 0.111 (2) Å and Φ_2_ = 186.4 (12) ° (Cremer & Pople, 1975). The C16—O2 distances in the indolone, in molecules A and B are 1.212 (2) ° and 1.208 (2) A °, respectively. The deviation of O2 from planarity seems to have considerably influenced, the torsion angles N2—C12–C16—O2 and C17—C15—C16—O2 (55.3 (2) ° and 7.2 (2)° in molecule A and -59.4 (2) ° and -1.1 (4)° in molecule B). The differences in these torsion angles may also be attributed due to O1—H1—N2 interaction in molecules A and B. The piperidine ring (N1/C2—C6) adopts a slightly twisted chair conformation in both molecules with the puckering parameters of Q = 0.668 (2) Å, Θ = 166.48 (2) ° and Φ = 22.0 (7) ° in molecule A, Q = 0.161 (2) Å, Θ = 13.97 (17)° and Φ = 203.5 (8) ° in molecule B (Cremer & Pople, 1975). Short contacts H26 ··· H8 (2.17 Å for molecule A and 2.20 Å for molecule B) result in substantial widening of the bond angles C7—C21—C26 (124.05I(17) ° for molecule A and 122.60 (18) ° for molecule B).

The structure features weak intra-molecular O—H···N, C—H···O interactions. The crystal structure features inter-molecular C—H···O and O—H···O interactions. The O1B—H1B···O3B interaction forms, inversely related dimers generating a graph set motif *R*_2_^2^(12). These dimers are further interconnected by C1A—H3···O3B and C1A—H2···*Cg*1 inter-molecular interactions (where *Cg*1 is the ring centroid of (C21A—C26A) as shown in Fig 2.

### 2. Experimental

A mixture of 1-methyl-3-[*E*-(4-chlorophenyl)methylidene]tetrahydro-2(1*H*)- pyridinone (1 mmol), ninhydrin (1 mmol) and proline (1 mmol) in methanol was refluxed for 3–4 h. After completion of the reaction as indicated by TLC the reaction mixture was poured into cold water. The solid precipitate obtained was filtered and dried. The product was purified by column chromatography using petroleum ether:ethylacetate mixture (90:10 V/V). Suitable crystals for the single-crystal-X-ray studies were obtained by recrystalizing the product from methanol. Yield: 57%, Melting point: 470–472 K.

### 3. Refinement

H atoms were placed at calculated positions and allowed to ride on their carrier atoms with C—H = 0.93–0.98 Å. *U*_iso_ = 1.2*U*_eq_(C) for CH_2_ and CH groups and *U*_iso_ = 1.5*U*_eq_(C) for CH_3_ group.

### Crystal data

**Table:**

C_26_H_25_ClN_2_O_3_	*F*(000) = 1888
*M**_r_* = 448.93	*D*_x_ = 1.358 Mg m^−^^3^
Monoclinic, *P*2_1_/*n*	Mo *K*α radiation, λ = 0.71073 Å
Hall symbol: -p 2yn	Cell parameters from 2000 reflections
*a* = 13.5839 (4) Å	θ = 2–31°
*b* = 11.3764 (4) Å	µ = 0.21 mm^−^^1^
*c* = 28.5382 (9) Å	*T* = 293 K
β = 95.120 (2)°	Block, colourless
*V* = 4392.6 (2) Å^3^	0.21 × 0.19 × 0.18 mm
*Z* = 8	

### Data collection

**Table:**

Bruker Kappa APEXII diffractometer	10365 independent reflections
Radiation source: fine-focus sealed tube	7088 reflections with *I* > 2σ(*I*)
Graphite monochromator	*R*_int_ = 0.041
Detector resolution: 0 pixels mm^-1^	θ_max_ = 27.8°, θ_min_ = 1.4°
ω and φ scans	*h* = −17→17
Absorption correction: multi-scan (*SADABS*; Sheldrick, 1996)	*k* = −14→14
*T*_min_ = 0.967, *T*_max_ = 0.974	*l* = −37→37
85408 measured reflections	

### Refinement

**Table:**

Refinement on *F*^2^	Primary atom site location: structure-invariant direct methods
Least-squares matrix: full	Secondary atom site location: difference Fourier map
*R*[*F*^2^ > 2σ(*F*^2^)] = 0.046	Hydrogen site location: inferred from neighbouring sites
*wR*(*F*^2^) = 0.123	H atoms treated by a mixture of independent and constrained refinement
*S* = 1.01	*w* = 1/[σ^2^(*F*_o_^2^) + (0.0445*P*)^2^ + 2.0326*P*] where *P* = (*F*_o_^2^ + 2*F*_c_^2^)/3
10365 reflections	(Δ/σ)_max_ < 0.001
591 parameters	Δρ_max_ = 0.33 e Å^−^^3^
0 restraints	Δρ_min_ = −0.38 e Å^−^^3^

### Special details

**Table:**

Geometry. All e.s.d.'s (except the e.s.d. in the dihedral angle between two l.s. planes) are estimated using the full covariance matrix. The cell e.s.d.'s are taken into account individually in the estimation of e.s.d.'s in distances, angles and torsion angles; correlations between e.s.d.'s in cell parameters are only used when they are defined by crystal symmetry. An approximate (isotropic) treatment of cell e.s.d.'s is used for estimating e.s.d.'s involving l.s. planes.
Refinement. Refinement of *F*^2^ against ALL reflections. The weighted *R*-factor *wR* and goodness of fit *S* are based on *F*^2^, conventional *R*-factors *R* are based on *F*, with *F* set to zero for negative *F*^2^. The threshold expression of *F*^2^ > σ (*F*^2^) is used only for calculating *R*-factors(gt) *etc*. and is not relevant to the choice of reflections for refinement. *R*-factors based on *F*.^2^ are statistically about twice as large as those based on *F*, and *R*-factors based on ALL data will be even larger.

### Fractional atomic coordinates and isotropic or equivalent isotropic displacement parameters (Å^2^)

**Table:**

	*x*	*y*	*z*	*U*_iso_*/*U*_eq_	
H2	0.2541 (19)	0.164 (2)	0.3395 (10)	0.088 (9)*	
H3	0.353 (2)	0.193 (3)	0.3711 (10)	0.106 (10)*	
C1A	0.2818 (2)	0.2028 (2)	0.37004 (10)	0.0629 (6)	
C2A	0.27703 (14)	0.38693 (18)	0.41290 (7)	0.0479 (5)	
H4	0.3484	0.3938	0.4175	0.057*	
H5	0.2550	0.3433	0.4392	0.057*	
C3A	0.23041 (14)	0.51019 (17)	0.41138 (6)	0.0452 (4)	
H3A	0.2476	0.5544	0.4404	0.054*	
C4A	0.26576 (14)	0.56995 (17)	0.36915 (7)	0.0453 (4)	
C5A	0.21563 (12)	0.50383 (15)	0.32732 (6)	0.0357 (4)	
C6A	0.27022 (13)	0.38621 (16)	0.32724 (6)	0.0402 (4)	
H6	0.2475	0.3409	0.2996	0.048*	
H7	0.3408	0.3991	0.3270	0.048*	
C7A	0.20490 (13)	0.57492 (16)	0.28073 (6)	0.0394 (4)	
H7A	0.2240	0.6563	0.2882	0.047*	
C8A	0.09354 (13)	0.57262 (16)	0.26891 (6)	0.0408 (4)	
H8A	0.0747	0.4980	0.2534	0.049*	
C9A	0.03576 (16)	0.67175 (19)	0.24399 (7)	0.0549 (5)	
H8	0.0302	0.6607	0.2101	0.066*	
H9	0.0663	0.7473	0.2515	0.066*	
C10A	−0.06550 (16)	0.6621 (2)	0.26367 (8)	0.0598 (6)	
H10A	−0.0870	0.7385	0.2739	0.072*	
H10B	−0.1145	0.6321	0.2399	0.072*	
C11A	−0.05198 (14)	0.5771 (2)	0.30546 (8)	0.0545 (5)	
H11A	−0.0843	0.6062	0.3321	0.065*	
H11B	−0.0774	0.4996	0.2970	0.065*	
C12A	0.10984 (12)	0.48544 (15)	0.34328 (6)	0.0350 (4)	
C13A	0.11777 (13)	0.50876 (16)	0.39756 (6)	0.0408 (4)	
C14A	0.06538 (13)	0.40713 (17)	0.41807 (6)	0.0433 (4)	
C15A	0.03857 (13)	0.32319 (17)	0.38446 (7)	0.0427 (4)	
C16A	0.06537 (13)	0.36182 (16)	0.33787 (6)	0.0391 (4)	
C17A	−0.00229 (14)	0.2168 (2)	0.39665 (8)	0.0562 (5)	
H17A	−0.0196	0.1600	0.3740	0.067*	
C18A	−0.01649 (16)	0.1976 (2)	0.44325 (9)	0.0693 (7)	
H18A	−0.0435	0.1268	0.4522	0.083*	
C19A	0.00885 (17)	0.2822 (3)	0.47666 (9)	0.0704 (7)	
H19A	−0.0022	0.2681	0.5079	0.084*	
C20A	0.05034 (15)	0.3872 (2)	0.46474 (7)	0.0572 (6)	
H20A	0.0679	0.4435	0.4876	0.069*	
C21A	0.26993 (14)	0.53118 (16)	0.24432 (6)	0.0412 (4)	
C22A	0.36564 (15)	0.57387 (19)	0.24535 (7)	0.0514 (5)	
H22A	0.3863	0.6312	0.2673	0.062*	
C23A	0.43091 (16)	0.5337 (2)	0.21480 (8)	0.0575 (5)	
H23A	0.4950	0.5631	0.2162	0.069*	
C24A	0.39997 (16)	0.44914 (18)	0.18209 (7)	0.0521 (5)	
C25A	0.30554 (17)	0.40619 (18)	0.17947 (7)	0.0531 (5)	
H25A	0.2848	0.3503	0.1569	0.064*	
C26A	0.24137 (15)	0.44680 (17)	0.21074 (7)	0.0475 (5)	
H26A	0.1775	0.4168	0.2092	0.057*	
N1A	0.24939 (11)	0.32397 (13)	0.36954 (5)	0.0406 (3)	
N2A	0.05550 (11)	0.57556 (13)	0.31548 (5)	0.0398 (3)	
O1A	0.07674 (12)	0.61834 (13)	0.40907 (5)	0.0595 (4)	
H1A	0.0688	0.6592	0.3854	0.089*	
O2A	0.04810 (11)	0.31079 (12)	0.30075 (5)	0.0524 (3)	
O3A	0.32204 (12)	0.65099 (15)	0.36775 (6)	0.0709 (5)	
Cl1	0.48368 (5)	0.39797 (6)	0.14410 (2)	0.07186 (18)	
H1	0.253 (2)	0.162 (2)	0.3931 (10)	0.087 (9)*	
C1B	0.8571 (3)	0.2820 (3)	0.19339 (9)	0.0890 (9)	
H10	0.8604	0.1977	0.1933	0.134*	
H11	0.7912	0.3063	0.1984	0.134*	
H12	0.9025	0.3123	0.2182	0.134*	
C2B	0.97308 (15)	0.27523 (19)	0.13400 (7)	0.0541 (5)	
H13	0.9672	0.1903	0.1338	0.065*	
H14	1.0282	0.2964	0.1564	0.065*	
C3B	0.99339 (14)	0.31785 (17)	0.08498 (7)	0.0484 (5)	
H3B	1.0541	0.2838	0.0746	0.058*	
C4B	0.99805 (14)	0.44975 (18)	0.08757 (7)	0.0492 (5)	
C5B	0.89285 (13)	0.48782 (16)	0.09453 (6)	0.0408 (4)	
C6B	0.87928 (16)	0.45404 (18)	0.14540 (7)	0.0502 (5)	
H15	0.9314	0.4885	0.1665	0.060*	
H16	0.8162	0.4825	0.1542	0.060*	
C7B	0.86807 (14)	0.61561 (17)	0.07776 (7)	0.0447 (4)	
H7B	0.9238	0.6443	0.0614	0.054*	
C8B	0.78183 (14)	0.59519 (18)	0.04095 (7)	0.0478 (5)	
H8B	0.7210	0.5887	0.0568	0.057*	
C9B	0.76111 (19)	0.6683 (2)	−0.00292 (8)	0.0667 (6)	
H17	0.8218	0.6983	−0.0139	0.080*	
H18	0.7177	0.7336	0.0025	0.080*	
C10B	0.7105 (2)	0.5801 (3)	−0.03822 (9)	0.0782 (8)	
H10C	0.6413	0.5998	−0.0452	0.094*	
H10D	0.7423	0.5803	−0.0673	0.094*	
C11B	0.72132 (17)	0.4603 (2)	−0.01442 (8)	0.0650 (6)	
H11C	0.6618	0.4385	−0.0001	0.078*	
H11D	0.7369	0.3996	−0.0365	0.078*	
C12B	0.83195 (13)	0.40302 (16)	0.06015 (6)	0.0397 (4)	
C13B	0.90496 (14)	0.30332 (17)	0.04781 (7)	0.0438 (4)	
C14B	0.84778 (16)	0.19125 (18)	0.05099 (7)	0.0508 (5)	
C15B	0.75861 (16)	0.2098 (2)	0.06915 (7)	0.0563 (5)	
C16B	0.74522 (14)	0.3361 (2)	0.07891 (7)	0.0520 (5)	
C17B	0.6951 (2)	0.1162 (3)	0.07601 (9)	0.0800 (8)	
H17B	0.6345	0.1291	0.0880	0.096*	
C18B	0.7238 (3)	0.0049 (3)	0.06471 (11)	0.0975 (12)	
H18B	0.6828	−0.0588	0.0694	0.117*	
C19B	0.8123 (3)	−0.0127 (2)	0.04657 (11)	0.0929 (11)	
H19B	0.8302	−0.0888	0.0389	0.111*	
C20B	0.8760 (2)	0.0783 (2)	0.03926 (8)	0.0689 (7)	
H20B	0.9361	0.0646	0.0269	0.083*	
C21B	0.84957 (14)	0.70415 (16)	0.11559 (7)	0.0438 (4)	
C22B	0.91994 (14)	0.78879 (18)	0.12870 (8)	0.0512 (5)	
H22B	0.9783	0.7905	0.1139	0.061*	
C23B	0.90558 (16)	0.87092 (18)	0.16324 (8)	0.0551 (5)	
H23B	0.9540	0.9266	0.1718	0.066*	
C24B	0.81938 (15)	0.86902 (17)	0.18455 (7)	0.0497 (5)	
C25B	0.74774 (15)	0.78766 (19)	0.17235 (7)	0.0521 (5)	
H25B	0.6892	0.7874	0.1870	0.063*	
C26B	0.76323 (15)	0.70569 (18)	0.13799 (7)	0.0503 (5)	
H26B	0.7145	0.6502	0.1297	0.060*	
N1B	0.88309 (13)	0.32669 (15)	0.14848 (6)	0.0501 (4)	
N2B	0.80391 (11)	0.48068 (15)	0.02110 (5)	0.0452 (4)	
O1B	0.93885 (12)	0.31807 (14)	0.00264 (5)	0.0630 (4)	
H1B	0.9168	0.3796	−0.0090	0.095*	
O2B	0.67501 (11)	0.38274 (17)	0.09432 (6)	0.0745 (5)	
O3B	1.06801 (11)	0.51295 (14)	0.08489 (7)	0.0746 (5)	
Cl2	0.80170 (5)	0.97008 (5)	0.22885 (2)	0.07313 (18)	

### Atomic displacement parameters (Å^2^)

**Table:**

	*U*^11^	*U*^22^	*U*^33^	*U*^12^	*U*^13^	*U*^23^
C1A	0.0832 (18)	0.0509 (13)	0.0561 (15)	0.0182 (12)	0.0141 (13)	0.0135 (11)
C2A	0.0424 (10)	0.0617 (13)	0.0384 (10)	0.0052 (9)	−0.0028 (8)	0.0062 (9)
C3A	0.0483 (10)	0.0522 (11)	0.0334 (9)	−0.0043 (9)	−0.0057 (8)	−0.0070 (8)
C4A	0.0429 (10)	0.0458 (11)	0.0458 (11)	−0.0044 (8)	−0.0038 (8)	−0.0032 (9)
C5A	0.0365 (9)	0.0366 (9)	0.0337 (9)	−0.0017 (7)	0.0016 (7)	0.0008 (7)
C6A	0.0375 (9)	0.0453 (10)	0.0383 (10)	0.0047 (8)	0.0056 (7)	0.0055 (8)
C7A	0.0474 (10)	0.0330 (9)	0.0375 (9)	−0.0033 (7)	0.0025 (8)	0.0024 (7)
C8A	0.0479 (10)	0.0388 (9)	0.0350 (9)	0.0001 (8)	−0.0002 (8)	0.0009 (7)
C9A	0.0659 (13)	0.0500 (12)	0.0469 (12)	0.0064 (10)	−0.0058 (10)	0.0101 (9)
C10A	0.0591 (13)	0.0643 (14)	0.0535 (13)	0.0189 (11)	−0.0098 (10)	0.0037 (11)
C11A	0.0427 (11)	0.0656 (13)	0.0545 (12)	0.0108 (10)	−0.0006 (9)	0.0048 (10)
C12A	0.0370 (9)	0.0366 (9)	0.0307 (8)	0.0031 (7)	0.0003 (7)	−0.0004 (7)
C13A	0.0458 (10)	0.0422 (10)	0.0340 (9)	0.0070 (8)	0.0015 (7)	−0.0031 (8)
C14A	0.0375 (9)	0.0547 (11)	0.0382 (10)	0.0101 (8)	0.0053 (8)	0.0048 (8)
C15A	0.0340 (9)	0.0488 (11)	0.0457 (11)	0.0022 (8)	0.0050 (8)	0.0059 (9)
C16A	0.0356 (9)	0.0420 (10)	0.0391 (10)	0.0017 (7)	0.0000 (7)	−0.0008 (8)
C17A	0.0430 (11)	0.0598 (13)	0.0659 (14)	−0.0051 (9)	0.0045 (10)	0.0123 (11)
C18A	0.0473 (12)	0.0860 (18)	0.0755 (17)	−0.0070 (12)	0.0106 (11)	0.0359 (15)
C19A	0.0529 (13)	0.107 (2)	0.0535 (14)	0.0044 (13)	0.0145 (11)	0.0282 (14)
C20A	0.0499 (11)	0.0825 (16)	0.0401 (11)	0.0112 (11)	0.0093 (9)	0.0062 (11)
C21A	0.0504 (11)	0.0383 (9)	0.0352 (9)	−0.0019 (8)	0.0053 (8)	0.0069 (8)
C22A	0.0543 (12)	0.0544 (12)	0.0458 (11)	−0.0083 (10)	0.0059 (9)	−0.0021 (9)
C23A	0.0516 (12)	0.0638 (14)	0.0585 (13)	−0.0041 (10)	0.0124 (10)	0.0061 (11)
C24A	0.0629 (13)	0.0488 (11)	0.0470 (11)	0.0094 (10)	0.0177 (10)	0.0113 (9)
C25A	0.0694 (14)	0.0457 (11)	0.0452 (11)	−0.0022 (10)	0.0106 (10)	−0.0013 (9)
C26A	0.0534 (11)	0.0449 (11)	0.0448 (11)	−0.0056 (9)	0.0077 (9)	0.0001 (9)
N1A	0.0435 (8)	0.0431 (8)	0.0352 (8)	0.0071 (7)	0.0035 (6)	0.0054 (7)
N2A	0.0413 (8)	0.0422 (8)	0.0353 (8)	0.0064 (6)	−0.0007 (6)	0.0024 (6)
O1A	0.0820 (10)	0.0513 (8)	0.0447 (8)	0.0204 (8)	0.0037 (8)	−0.0089 (7)
O2A	0.0661 (9)	0.0468 (8)	0.0429 (8)	−0.0086 (7)	−0.0028 (6)	−0.0062 (6)
O3A	0.0751 (11)	0.0716 (11)	0.0636 (10)	−0.0352 (9)	−0.0071 (8)	−0.0027 (8)
Cl1	0.0799 (4)	0.0717 (4)	0.0688 (4)	0.0167 (3)	0.0327 (3)	0.0072 (3)
C1B	0.136 (3)	0.0754 (18)	0.0601 (16)	0.0138 (17)	0.0306 (16)	0.0153 (14)
C2B	0.0559 (12)	0.0466 (11)	0.0577 (13)	0.0071 (9)	−0.0065 (10)	−0.0009 (10)
C3B	0.0371 (10)	0.0468 (11)	0.0621 (13)	0.0059 (8)	0.0082 (9)	0.0007 (9)
C4B	0.0369 (10)	0.0491 (11)	0.0609 (13)	0.0008 (9)	0.0005 (9)	0.0003 (10)
C5B	0.0365 (9)	0.0420 (10)	0.0436 (10)	0.0026 (7)	0.0018 (8)	−0.0028 (8)
C6B	0.0578 (12)	0.0506 (11)	0.0409 (10)	0.0053 (9)	−0.0031 (9)	−0.0067 (9)
C7B	0.0409 (10)	0.0455 (10)	0.0480 (11)	0.0061 (8)	0.0052 (8)	0.0006 (8)
C8B	0.0455 (10)	0.0547 (12)	0.0435 (11)	0.0130 (9)	0.0049 (8)	−0.0028 (9)
C9B	0.0735 (15)	0.0728 (16)	0.0533 (13)	0.0269 (13)	0.0033 (11)	0.0065 (12)
C10B	0.0857 (18)	0.098 (2)	0.0486 (13)	0.0361 (15)	−0.0093 (12)	−0.0017 (13)
C11B	0.0630 (14)	0.0873 (17)	0.0428 (12)	0.0138 (12)	−0.0067 (10)	−0.0161 (12)
C12B	0.0353 (9)	0.0482 (10)	0.0363 (9)	0.0007 (8)	0.0063 (7)	−0.0041 (8)
C13B	0.0460 (10)	0.0465 (11)	0.0407 (10)	0.0027 (8)	0.0132 (8)	−0.0012 (8)
C14B	0.0658 (13)	0.0490 (12)	0.0365 (10)	−0.0062 (10)	−0.0011 (9)	−0.0019 (9)
C15B	0.0621 (13)	0.0655 (14)	0.0402 (11)	−0.0195 (11)	−0.0012 (9)	0.0016 (10)
C16B	0.0410 (10)	0.0768 (15)	0.0389 (10)	−0.0083 (10)	0.0068 (8)	−0.0053 (10)
C17B	0.0836 (18)	0.097 (2)	0.0556 (15)	−0.0411 (16)	−0.0118 (13)	0.0175 (14)
C18B	0.131 (3)	0.076 (2)	0.077 (2)	−0.053 (2)	−0.038 (2)	0.0258 (16)
C19B	0.146 (3)	0.0473 (14)	0.0761 (19)	−0.0202 (18)	−0.043 (2)	0.0091 (13)
C20B	0.0987 (19)	0.0507 (13)	0.0542 (14)	0.0024 (13)	−0.0109 (13)	−0.0009 (11)
C21B	0.0428 (10)	0.0394 (10)	0.0479 (11)	0.0073 (8)	−0.0022 (8)	0.0024 (8)
C22B	0.0422 (10)	0.0484 (11)	0.0624 (13)	0.0042 (9)	0.0023 (9)	0.0035 (10)
C23B	0.0539 (12)	0.0446 (11)	0.0645 (14)	−0.0014 (9)	−0.0084 (10)	−0.0027 (10)
C24B	0.0556 (12)	0.0412 (10)	0.0501 (12)	0.0096 (9)	−0.0066 (9)	−0.0023 (9)
C25B	0.0475 (11)	0.0561 (12)	0.0523 (12)	0.0066 (9)	0.0020 (9)	−0.0069 (10)
C26B	0.0451 (11)	0.0499 (11)	0.0553 (12)	0.0006 (9)	0.0006 (9)	−0.0071 (9)
N1B	0.0628 (10)	0.0477 (9)	0.0400 (9)	0.0043 (8)	0.0063 (8)	0.0010 (7)
N2B	0.0442 (9)	0.0554 (10)	0.0364 (8)	0.0084 (7)	0.0048 (7)	−0.0028 (7)
O1B	0.0782 (10)	0.0615 (10)	0.0542 (9)	0.0205 (8)	0.0330 (8)	0.0069 (7)
O2B	0.0466 (8)	0.1099 (14)	0.0703 (11)	−0.0027 (9)	0.0236 (8)	−0.0088 (10)
O3B	0.0399 (8)	0.0588 (10)	0.1244 (16)	−0.0073 (7)	0.0032 (9)	0.0015 (10)
Cl2	0.0842 (4)	0.0606 (4)	0.0737 (4)	0.0030 (3)	0.0022 (3)	−0.0226 (3)

### Geometric parameters (Å, º)

**Table:**

C1A—N1A	1.447 (3)	C1B—N1B	1.451 (3)
C1A—H2	1.02 (3)	C1B—H10	0.9600
C1A—H3	0.96 (3)	C1B—H11	0.9600
C1A—H1	0.92 (3)	C1B—H12	0.9600
C2A—N1A	1.450 (2)	C2B—N1B	1.448 (3)
C2A—C3A	1.538 (3)	C2B—C3B	1.529 (3)
C2A—H4	0.9700	C2B—H13	0.9700
C2A—H5	0.9700	C2B—H14	0.9700
C3A—C4A	1.500 (3)	C3B—C4B	1.503 (3)
C3A—C13A	1.546 (3)	C3B—C13B	1.539 (3)
C3A—H3A	0.9800	C3B—H3B	0.9800
C4A—O3A	1.201 (2)	C4B—O3B	1.200 (2)
C4A—C5A	1.520 (2)	C4B—C5B	1.523 (3)
C5A—C6A	1.530 (2)	C5B—C6B	1.529 (3)
C5A—C7A	1.552 (2)	C5B—C7B	1.558 (3)
C5A—C12A	1.560 (2)	C5B—C12B	1.560 (3)
C6A—N1A	1.449 (2)	C6B—N1B	1.452 (3)
C6A—H6	0.9700	C6B—H15	0.9700
C6A—H7	0.9700	C6B—H16	0.9700
C7A—C21A	1.507 (3)	C7B—C21B	1.514 (3)
C7A—C8A	1.520 (3)	C7B—C8B	1.520 (3)
C7A—H7A	0.9800	C7B—H7B	0.9800
C8A—N2A	1.469 (2)	C8B—N2B	1.462 (2)
C8A—C9A	1.515 (3)	C8B—C9B	1.508 (3)
C8A—H8A	0.9800	C8B—H8B	0.9800
C9A—C10A	1.535 (3)	C9B—C10B	1.539 (4)
C9A—H8	0.9700	C9B—H17	0.9700
C9A—H9	0.9700	C9B—H18	0.9700
C10A—C11A	1.534 (3)	C10B—C11B	1.524 (4)
C10A—H10A	0.9700	C10B—H10C	0.9700
C10A—H10B	0.9700	C10B—H10D	0.9700
C11A—N2A	1.462 (2)	C11B—N2B	1.462 (3)
C11A—H11A	0.9700	C11B—H11C	0.9700
C11A—H11B	0.9700	C11B—H11D	0.9700
C12A—N2A	1.456 (2)	C12B—N2B	1.446 (2)
C12A—C16A	1.533 (2)	C12B—C16B	1.538 (3)
C12A—C13A	1.566 (2)	C12B—C13B	1.567 (3)
C13A—O1A	1.416 (2)	C13B—O1B	1.417 (2)
C13A—C14A	1.503 (3)	C13B—C14B	1.500 (3)
C14A—C15A	1.379 (3)	C14B—C15B	1.376 (3)
C14A—C20A	1.384 (3)	C14B—C20B	1.391 (3)
C15A—C17A	1.389 (3)	C15B—C17B	1.395 (3)
C15A—C16A	1.476 (3)	C15B—C16B	1.478 (3)
C16A—O2A	1.212 (2)	C16B—O2B	1.208 (2)
C17A—C18A	1.378 (3)	C17B—C18B	1.373 (5)
C17A—H17A	0.9300	C17B—H17B	0.9300
C18A—C19A	1.376 (4)	C18B—C19B	1.366 (5)
C18A—H18A	0.9300	C18B—H18B	0.9300
C19A—C20A	1.377 (3)	C19B—C20B	1.377 (4)
C19A—H19A	0.9300	C19B—H19B	0.9300
C20A—H20A	0.9300	C20B—H20B	0.9300
C21A—C22A	1.386 (3)	C21B—C22B	1.385 (3)
C21A—C26A	1.387 (3)	C21B—C26B	1.385 (3)
C22A—C23A	1.376 (3)	C22B—C23B	1.385 (3)
C22A—H22A	0.9300	C22B—H22B	0.9300
C23A—C24A	1.379 (3)	C23B—C24B	1.367 (3)
C23A—H23A	0.9300	C23B—H23B	0.9300
C24A—C25A	1.368 (3)	C24B—C25B	1.365 (3)
C24A—Cl1	1.740 (2)	C24B—Cl2	1.741 (2)
C25A—C26A	1.382 (3)	C25B—C26B	1.383 (3)
C25A—H25A	0.9300	C25B—H25B	0.9300
C26A—H26A	0.9300	C26B—H26B	0.9300
O1A—H1A	0.8200	O1B—H1B	0.8200
			
N1A—C1A—H2	108.4 (15)	N1B—C1B—H10	109.5
N1A—C1A—H3	114.2 (19)	N1B—C1B—H11	109.5
H2—C1A—H3	106 (2)	H10—C1B—H11	109.5
N1A—C1A—H1	109.7 (17)	N1B—C1B—H12	109.5
H2—C1A—H1	104 (2)	H10—C1B—H12	109.5
H3—C1A—H1	114 (2)	H11—C1B—H12	109.5
N1A—C2A—C3A	110.60 (15)	N1B—C2B—C3B	111.03 (16)
N1A—C2A—H4	109.5	N1B—C2B—H13	109.4
C3A—C2A—H4	109.5	C3B—C2B—H13	109.4
N1A—C2A—H5	109.5	N1B—C2B—H14	109.4
C3A—C2A—H5	109.5	C3B—C2B—H14	109.4
H4—C2A—H5	108.1	H13—C2B—H14	108.0
C4A—C3A—C2A	105.99 (16)	C4B—C3B—C2B	106.37 (17)
C4A—C3A—C13A	100.39 (15)	C4B—C3B—C13B	99.67 (15)
C2A—C3A—C13A	113.17 (16)	C2B—C3B—C13B	113.77 (16)
C4A—C3A—H3A	112.2	C4B—C3B—H3B	112.1
C2A—C3A—H3A	112.2	C2B—C3B—H3B	112.1
C13A—C3A—H3A	112.2	C13B—C3B—H3B	112.1
O3A—C4A—C3A	128.70 (18)	O3B—C4B—C3B	128.70 (18)
O3A—C4A—C5A	126.59 (18)	O3B—C4B—C5B	126.56 (19)
C3A—C4A—C5A	104.70 (15)	C3B—C4B—C5B	104.74 (15)
C4A—C5A—C6A	104.52 (14)	C4B—C5B—C6B	104.26 (15)
C4A—C5A—C7A	114.60 (15)	C4B—C5B—C7B	113.88 (15)
C6A—C5A—C7A	117.58 (14)	C6B—C5B—C7B	119.04 (15)
C4A—C5A—C12A	101.36 (14)	C4B—C5B—C12B	101.13 (14)
C6A—C5A—C12A	110.02 (14)	C6B—C5B—C12B	109.86 (15)
C7A—C5A—C12A	107.55 (13)	C7B—C5B—C12B	107.22 (14)
N1A—C6A—C5A	107.18 (14)	N1B—C6B—C5B	107.56 (15)
N1A—C6A—H6	110.3	N1B—C6B—H15	110.2
C5A—C6A—H6	110.3	C5B—C6B—H15	110.2
N1A—C6A—H7	110.3	N1B—C6B—H16	110.2
C5A—C6A—H7	110.3	C5B—C6B—H16	110.2
H6—C6A—H7	108.5	H15—C6B—H16	108.5
C21A—C7A—C8A	118.30 (15)	C21B—C7B—C8B	115.33 (15)
C21A—C7A—C5A	113.74 (15)	C21B—C7B—C5B	116.54 (16)
C8A—C7A—C5A	101.33 (14)	C8B—C7B—C5B	101.64 (15)
C21A—C7A—H7A	107.6	C21B—C7B—H7B	107.6
C8A—C7A—H7A	107.6	C8B—C7B—H7B	107.6
C5A—C7A—H7A	107.6	C5B—C7B—H7B	107.6
N2A—C8A—C9A	101.46 (15)	N2B—C8B—C9B	101.49 (16)
N2A—C8A—C7A	102.85 (14)	N2B—C8B—C7B	103.40 (15)
C9A—C8A—C7A	123.70 (16)	C9B—C8B—C7B	124.37 (19)
N2A—C8A—H8A	109.2	N2B—C8B—H8B	108.8
C9A—C8A—H8A	109.2	C9B—C8B—H8B	108.8
C7A—C8A—H8A	109.2	C7B—C8B—H8B	108.8
C8A—C9A—C10A	102.64 (16)	C8B—C9B—C10B	102.9 (2)
C8A—C9A—H8	111.2	C8B—C9B—H17	111.2
C10A—C9A—H8	111.2	C10B—C9B—H17	111.2
C8A—C9A—H9	111.2	C8B—C9B—H18	111.2
C10A—C9A—H9	111.2	C10B—C9B—H18	111.2
H8—C9A—H9	109.2	H17—C9B—H18	109.1
C11A—C10A—C9A	106.28 (16)	C11B—C10B—C9B	105.76 (18)
C11A—C10A—H10A	110.5	C11B—C10B—H10C	110.6
C9A—C10A—H10A	110.5	C9B—C10B—H10C	110.6
C11A—C10A—H10B	110.5	C11B—C10B—H10D	110.6
C9A—C10A—H10B	110.5	C9B—C10B—H10D	110.6
H10A—C10A—H10B	108.7	H10C—C10B—H10D	108.7
N2A—C11A—C10A	102.00 (17)	N2B—C11B—C10B	101.8 (2)
N2A—C11A—H11A	111.4	N2B—C11B—H11C	111.4
C10A—C11A—H11A	111.4	C10B—C11B—H11C	111.4
N2A—C11A—H11B	111.4	N2B—C11B—H11D	111.4
C10A—C11A—H11B	111.4	C10B—C11B—H11D	111.4
H11A—C11A—H11B	109.2	H11C—C11B—H11D	109.3
N2A—C12A—C16A	114.52 (14)	N2B—C12B—C16B	114.40 (15)
N2A—C12A—C5A	100.48 (13)	N2B—C12B—C5B	101.26 (14)
C16A—C12A—C5A	117.31 (14)	C16B—C12B—C5B	117.83 (15)
N2A—C12A—C13A	113.98 (14)	N2B—C12B—C13B	113.41 (14)
C16A—C12A—C13A	104.28 (14)	C16B—C12B—C13B	103.87 (16)
C5A—C12A—C13A	106.40 (13)	C5B—C12B—C13B	106.16 (14)
O1A—C13A—C14A	112.15 (15)	O1B—C13B—C14B	111.57 (16)
O1A—C13A—C3A	109.43 (16)	O1B—C13B—C3B	108.50 (16)
C14A—C13A—C3A	113.63 (15)	C14B—C13B—C3B	115.23 (16)
O1A—C13A—C12A	112.64 (14)	O1B—C13B—C12B	112.47 (15)
C14A—C13A—C12A	105.17 (14)	C14B—C13B—C12B	105.08 (15)
C3A—C13A—C12A	103.49 (14)	C3B—C13B—C12B	103.77 (15)
C15A—C14A—C20A	120.2 (2)	C15B—C14B—C20B	120.2 (2)
C15A—C14A—C13A	111.51 (16)	C15B—C14B—C13B	111.76 (18)
C20A—C14A—C13A	128.05 (19)	C20B—C14B—C13B	128.0 (2)
C14A—C15A—C17A	121.06 (19)	C14B—C15B—C17B	120.9 (2)
C14A—C15A—C16A	110.43 (16)	C14B—C15B—C16B	110.32 (18)
C17A—C15A—C16A	128.41 (19)	C17B—C15B—C16B	128.8 (2)
O2A—C16A—C15A	126.89 (17)	O2B—C16B—C15B	127.6 (2)
O2A—C16A—C12A	124.82 (17)	O2B—C16B—C12B	124.3 (2)
C15A—C16A—C12A	108.00 (15)	C15B—C16B—C12B	107.73 (17)
C18A—C17A—C15A	118.2 (2)	C18B—C17B—C15B	118.6 (3)
C18A—C17A—H17A	120.9	C18B—C17B—H17B	120.7
C15A—C17A—H17A	120.9	C15B—C17B—H17B	120.7
C19A—C18A—C17A	120.7 (2)	C19B—C18B—C17B	120.2 (3)
C19A—C18A—H18A	119.7	C19B—C18B—H18B	119.9
C17A—C18A—H18A	119.7	C17B—C18B—H18B	119.9
C18A—C19A—C20A	121.2 (2)	C18B—C19B—C20B	122.3 (3)
C18A—C19A—H19A	119.4	C18B—C19B—H19B	118.9
C20A—C19A—H19A	119.4	C20B—C19B—H19B	118.9
C19A—C20A—C14A	118.6 (2)	C19B—C20B—C14B	117.9 (3)
C19A—C20A—H20A	120.7	C19B—C20B—H20B	121.1
C14A—C20A—H20A	120.7	C14B—C20B—H20B	121.1
C22A—C21A—C26A	117.46 (18)	C22B—C21B—C26B	117.33 (18)
C22A—C21A—C7A	118.44 (17)	C22B—C21B—C7B	120.06 (18)
C26A—C21A—C7A	124.05 (17)	C26B—C21B—C7B	122.60 (18)
C23A—C22A—C21A	121.8 (2)	C21B—C22B—C23B	121.60 (19)
C23A—C22A—H22A	119.1	C21B—C22B—H22B	119.2
C21A—C22A—H22A	119.1	C23B—C22B—H22B	119.2
C22A—C23A—C24A	119.1 (2)	C24B—C23B—C22B	119.13 (19)
C22A—C23A—H23A	120.5	C24B—C23B—H23B	120.4
C24A—C23A—H23A	120.5	C22B—C23B—H23B	120.4
C25A—C24A—C23A	120.89 (19)	C25B—C24B—C23B	121.11 (19)
C25A—C24A—Cl1	120.53 (17)	C25B—C24B—Cl2	119.43 (17)
C23A—C24A—Cl1	118.58 (17)	C23B—C24B—Cl2	119.45 (16)
C24A—C25A—C26A	119.2 (2)	C24B—C25B—C26B	119.2 (2)
C24A—C25A—H25A	120.4	C24B—C25B—H25B	120.4
C26A—C25A—H25A	120.4	C26B—C25B—H25B	120.4
C25A—C26A—C21A	121.61 (19)	C25B—C26B—C21B	121.63 (19)
C25A—C26A—H26A	119.2	C25B—C26B—H26B	119.2
C21A—C26A—H26A	119.2	C21B—C26B—H26B	119.2
C1A—N1A—C6A	113.07 (16)	C2B—N1B—C1B	112.85 (19)
C1A—N1A—C2A	114.00 (17)	C2B—N1B—C6B	114.35 (17)
C6A—N1A—C2A	114.49 (15)	C1B—N1B—C6B	113.15 (18)
C12A—N2A—C11A	124.31 (15)	C12B—N2B—C11B	124.78 (17)
C12A—N2A—C8A	105.94 (13)	C12B—N2B—C8B	107.15 (14)
C11A—N2A—C8A	104.43 (14)	C11B—N2B—C8B	103.81 (15)
C13A—O1A—H1A	109.5	C13B—O1B—H1B	109.5
			
N1A—C2A—C3A—C4A	−57.63 (19)	N1B—C2B—C3B—C4B	57.2 (2)
N1A—C2A—C3A—C13A	51.4 (2)	N1B—C2B—C3B—C13B	−51.5 (2)
C2A—C3A—C4A—O3A	−110.9 (2)	C2B—C3B—C4B—O3B	112.0 (3)
C13A—C3A—C4A—O3A	131.2 (2)	C13B—C3B—C4B—O3B	−129.5 (2)
C2A—C3A—C4A—C5A	68.25 (17)	C2B—C3B—C4B—C5B	−67.91 (19)
C13A—C3A—C4A—C5A	−49.70 (18)	C13B—C3B—C4B—C5B	50.53 (18)
O3A—C4A—C5A—C6A	105.8 (2)	O3B—C4B—C5B—C6B	−107.0 (2)
C3A—C4A—C5A—C6A	−73.31 (17)	C3B—C4B—C5B—C6B	72.93 (19)
O3A—C4A—C5A—C7A	−24.3 (3)	O3B—C4B—C5B—C7B	24.3 (3)
C3A—C4A—C5A—C7A	156.55 (15)	C3B—C4B—C5B—C7B	−155.75 (16)
O3A—C4A—C5A—C12A	−139.8 (2)	O3B—C4B—C5B—C12B	139.0 (2)
C3A—C4A—C5A—C12A	41.07 (17)	C3B—C4B—C5B—C12B	−41.10 (19)
C4A—C5A—C6A—N1A	65.78 (17)	C4B—C5B—C6B—N1B	−65.79 (19)
C7A—C5A—C6A—N1A	−165.87 (14)	C7B—C5B—C6B—N1B	165.98 (16)
C12A—C5A—C6A—N1A	−42.34 (18)	C12B—C5B—C6B—N1B	41.9 (2)
C4A—C5A—C7A—C21A	110.27 (18)	C4B—C5B—C7B—C21B	−112.93 (19)
C6A—C5A—C7A—C21A	−13.1 (2)	C6B—C5B—C7B—C21B	10.7 (2)
C12A—C5A—C7A—C21A	−137.89 (15)	C12B—C5B—C7B—C21B	136.07 (16)
C4A—C5A—C7A—C8A	−121.68 (16)	C4B—C5B—C7B—C8B	120.83 (17)
C6A—C5A—C7A—C8A	114.94 (16)	C6B—C5B—C7B—C8B	−115.54 (18)
C12A—C5A—C7A—C8A	−9.84 (17)	C12B—C5B—C7B—C8B	9.83 (18)
C21A—C7A—C8A—N2A	158.96 (15)	C21B—C7B—C8B—N2B	−159.34 (16)
C5A—C7A—C8A—N2A	33.91 (16)	C5B—C7B—C8B—N2B	−32.31 (18)
C21A—C7A—C8A—C9A	−87.6 (2)	C21B—C7B—C8B—C9B	86.4 (2)
C5A—C7A—C8A—C9A	147.30 (17)	C5B—C7B—C8B—C9B	−146.57 (19)
N2A—C8A—C9A—C10A	−36.18 (19)	N2B—C8B—C9B—C10B	34.9 (2)
C7A—C8A—C9A—C10A	−150.26 (18)	C7B—C8B—C9B—C10B	150.1 (2)
C8A—C9A—C10A—C11A	11.7 (2)	C8B—C9B—C10B—C11B	−8.9 (2)
C9A—C10A—C11A—N2A	17.2 (2)	C9B—C10B—C11B—N2B	−20.4 (2)
C4A—C5A—C12A—N2A	102.79 (14)	C4B—C5B—C12B—N2B	−103.29 (15)
C6A—C5A—C12A—N2A	−147.00 (14)	C6B—C5B—C12B—N2B	146.95 (14)
C7A—C5A—C12A—N2A	−17.79 (16)	C7B—C5B—C12B—N2B	16.24 (17)
C4A—C5A—C12A—C16A	−132.43 (15)	C4B—C5B—C12B—C16B	131.17 (18)
C6A—C5A—C12A—C16A	−22.2 (2)	C6B—C5B—C12B—C16B	21.4 (2)
C7A—C5A—C12A—C16A	106.99 (16)	C7B—C5B—C12B—C16B	−109.30 (19)
C4A—C5A—C12A—C13A	−16.24 (17)	C4B—C5B—C12B—C13B	15.37 (18)
C6A—C5A—C12A—C13A	93.97 (16)	C6B—C5B—C12B—C13B	−94.39 (17)
C7A—C5A—C12A—C13A	−136.82 (14)	C7B—C5B—C12B—C13B	134.90 (15)
C4A—C3A—C13A—O1A	−83.08 (17)	C4B—C3B—C13B—O1B	81.23 (18)
C2A—C3A—C13A—O1A	164.40 (15)	C2B—C3B—C13B—O1B	−165.97 (16)
C4A—C3A—C13A—C14A	150.71 (16)	C4B—C3B—C13B—C14B	−152.87 (17)
C2A—C3A—C13A—C14A	38.2 (2)	C2B—C3B—C13B—C14B	−40.1 (2)
C4A—C3A—C13A—C12A	37.21 (17)	C4B—C3B—C13B—C12B	−38.57 (18)
C2A—C3A—C13A—C12A	−75.31 (18)	C2B—C3B—C13B—C12B	74.23 (19)
N2A—C12A—C13A—O1A	−4.5 (2)	N2B—C12B—C13B—O1B	7.6 (2)
C16A—C12A—C13A—O1A	−130.12 (16)	C16B—C12B—C13B—O1B	132.37 (17)
C5A—C12A—C13A—O1A	105.25 (17)	C5B—C12B—C13B—O1B	−102.72 (18)
N2A—C12A—C13A—C14A	117.90 (16)	N2B—C12B—C13B—C14B	−113.99 (17)
C16A—C12A—C13A—C14A	−7.68 (17)	C16B—C12B—C13B—C14B	10.80 (18)
C5A—C12A—C13A—C14A	−132.32 (14)	C5B—C12B—C13B—C14B	135.71 (15)
N2A—C12A—C13A—C3A	−122.62 (15)	N2B—C12B—C13B—C3B	124.64 (16)
C16A—C12A—C13A—C3A	111.80 (15)	C16B—C12B—C13B—C3B	−110.56 (16)
C5A—C12A—C13A—C3A	−12.83 (17)	C5B—C12B—C13B—C3B	14.34 (18)
O1A—C13A—C14A—C15A	129.46 (17)	O1B—C13B—C14B—C15B	−130.14 (18)
C3A—C13A—C14A—C15A	−105.78 (18)	C3B—C13B—C14B—C15B	105.6 (2)
C12A—C13A—C14A—C15A	6.71 (19)	C12B—C13B—C14B—C15B	−8.0 (2)
O1A—C13A—C14A—C20A	−56.2 (2)	O1B—C13B—C14B—C20B	51.6 (3)
C3A—C13A—C14A—C20A	68.6 (2)	C3B—C13B—C14B—C20B	−72.7 (3)
C12A—C13A—C14A—C20A	−178.91 (18)	C12B—C13B—C14B—C20B	173.8 (2)
C20A—C14A—C15A—C17A	−1.1 (3)	C20B—C14B—C15B—C17B	−0.1 (3)
C13A—C14A—C15A—C17A	173.81 (17)	C13B—C14B—C15B—C17B	−178.52 (19)
C20A—C14A—C15A—C16A	−177.66 (17)	C20B—C14B—C15B—C16B	179.84 (19)
C13A—C14A—C15A—C16A	−2.8 (2)	C13B—C14B—C15B—C16B	1.5 (2)
C14A—C15A—C16A—O2A	−176.55 (18)	C14B—C15B—C16B—O2B	178.9 (2)
C17A—C15A—C16A—O2A	7.2 (3)	C17B—C15B—C16B—O2B	−1.1 (4)
C14A—C15A—C16A—C12A	−2.5 (2)	C14B—C15B—C16B—C12B	5.9 (2)
C17A—C15A—C16A—C12A	−178.77 (18)	C17B—C15B—C16B—C12B	−174.1 (2)
N2A—C12A—C16A—O2A	55.3 (2)	N2B—C12B—C16B—O2B	−59.4 (3)
C5A—C12A—C16A—O2A	−62.1 (2)	C5B—C12B—C16B—O2B	59.4 (3)
C13A—C12A—C16A—O2A	−179.44 (17)	C13B—C12B—C16B—O2B	176.43 (19)
N2A—C12A—C16A—C15A	−118.90 (16)	N2B—C12B—C16B—C15B	113.88 (18)
C5A—C12A—C16A—C15A	123.68 (16)	C5B—C12B—C16B—C15B	−127.31 (17)
C13A—C12A—C16A—C15A	6.34 (17)	C13B—C12B—C16B—C15B	−10.3 (2)
C14A—C15A—C17A—C18A	0.7 (3)	C14B—C15B—C17B—C18B	0.6 (3)
C16A—C15A—C17A—C18A	176.67 (19)	C16B—C15B—C17B—C18B	−179.3 (2)
C15A—C17A—C18A—C19A	0.3 (3)	C15B—C17B—C18B—C19B	−0.8 (4)
C17A—C18A—C19A—C20A	−1.0 (4)	C17B—C18B—C19B—C20B	0.5 (4)
C18A—C19A—C20A—C14A	0.7 (3)	C18B—C19B—C20B—C14B	0.0 (4)
C15A—C14A—C20A—C19A	0.4 (3)	C15B—C14B—C20B—C19B	−0.2 (3)
C13A—C14A—C20A—C19A	−173.59 (19)	C13B—C14B—C20B—C19B	177.9 (2)
C8A—C7A—C21A—C22A	154.36 (17)	C8B—C7B—C21B—C22B	−134.64 (19)
C5A—C7A—C21A—C22A	−86.9 (2)	C5B—C7B—C21B—C22B	106.3 (2)
C8A—C7A—C21A—C26A	−28.4 (3)	C8B—C7B—C21B—C26B	44.6 (3)
C5A—C7A—C21A—C26A	90.4 (2)	C5B—C7B—C21B—C26B	−74.4 (2)
C26A—C21A—C22A—C23A	−0.8 (3)	C26B—C21B—C22B—C23B	0.9 (3)
C7A—C21A—C22A—C23A	176.65 (19)	C7B—C21B—C22B—C23B	−179.83 (18)
C21A—C22A—C23A—C24A	0.4 (3)	C21B—C22B—C23B—C24B	−0.7 (3)
C22A—C23A—C24A—C25A	0.7 (3)	C22B—C23B—C24B—C25B	0.1 (3)
C22A—C23A—C24A—Cl1	−179.32 (16)	C22B—C23B—C24B—Cl2	178.72 (16)
C23A—C24A—C25A—C26A	−1.3 (3)	C23B—C24B—C25B—C26B	0.3 (3)
Cl1—C24A—C25A—C26A	178.69 (15)	Cl2—C24B—C25B—C26B	−178.36 (16)
C24A—C25A—C26A—C21A	0.9 (3)	C24B—C25B—C26B—C21B	−0.1 (3)
C22A—C21A—C26A—C25A	0.2 (3)	C22B—C21B—C26B—C25B	−0.5 (3)
C7A—C21A—C26A—C25A	−177.14 (18)	C7B—C21B—C26B—C25B	−179.77 (18)
C5A—C6A—N1A—C1A	169.67 (19)	C3B—C2B—N1B—C1B	175.8 (2)
C5A—C6A—N1A—C2A	−57.50 (19)	C3B—C2B—N1B—C6B	−53.0 (2)
C3A—C2A—N1A—C1A	−173.92 (19)	C5B—C6B—N1B—C2B	57.2 (2)
C3A—C2A—N1A—C6A	53.7 (2)	C5B—C6B—N1B—C1B	−171.7 (2)
C16A—C12A—N2A—C11A	34.4 (2)	C16B—C12B—N2B—C11B	−31.6 (2)
C5A—C12A—N2A—C11A	161.08 (17)	C5B—C12B—N2B—C11B	−159.41 (17)
C13A—C12A—N2A—C11A	−85.6 (2)	C13B—C12B—N2B—C11B	87.3 (2)
C16A—C12A—N2A—C8A	−86.16 (17)	C16B—C12B—N2B—C8B	89.63 (19)
C5A—C12A—N2A—C8A	40.51 (16)	C5B—C12B—N2B—C8B	−38.18 (17)
C13A—C12A—N2A—C8A	153.87 (15)	C13B—C12B—N2B—C8B	−151.47 (15)
C10A—C11A—N2A—C12A	−162.38 (16)	C10B—C11B—N2B—C12B	166.35 (17)
C10A—C11A—N2A—C8A	−41.11 (19)	C10B—C11B—N2B—C8B	43.6 (2)
C9A—C8A—N2A—C12A	−177.77 (14)	C9B—C8B—N2B—C12B	176.22 (16)
C7A—C8A—N2A—C12A	−48.94 (17)	C7B—C8B—N2B—C12B	46.38 (19)
C9A—C8A—N2A—C11A	49.47 (18)	C9B—C8B—N2B—C11B	−50.1 (2)
C7A—C8A—N2A—C11A	178.30 (15)	C7B—C8B—N2B—C11B	−179.94 (17)

### Hydrogen-bond geometry (Å, º)

*Cg*1 is the centroid of the C21A–C26A ring.

**Table:**

*D*—H···*A*	*D*—H	H···*A*	*D*···*A*	*D*—H···*A*
O1*A*—H1*A*···N2*A*	0.82	2.20	2.705 (2)	120
O1*B*—H1*B*···N2*B*	0.82	2.16	2.689 (2)	123
C8*A*—H8*A*···O2*A*	0.98	2.57	3.190 (2)	122
O1*B*—H1*B*···O3*B*^i^	0.82	2.51	3.147 (2)	135
C1*A*—H3···O3*B*^ii^	0.96 (3)	2.59 (3)	3.164 (3)	118 (2)
C1*A*—H2···*Cg*1^iii^	1.02 (3)	2.70 (3)	3.648 (3)	155 (2)

Symmetry codes: (i) −*x*+2, −*y*+1, −*z*; (ii) −*x*+3/2, *y*−1/2, −*z*+1/2; (iii) −*x*+1/2, *y*−1/2, −*z*+1/2.
